# Limiting systemic endocrine overtreatment in postmenopausal breast cancer patients with an ultralow classification of the 70-gene signature

**DOI:** 10.1007/s10549-022-06618-z

**Published:** 2022-05-19

**Authors:** M. Opdam, V. van der Noort, M. Kleijn, A. Glas, I. Mandjes, S. Kleiterp, F. S. Hilbers, D. T. Kruger, A. D. Bins, P. C. de Jong, P. P. J. B. M. Schiphorst, T. van Dalen, B. Flameling, R. C. Rietbroek, A. Beeker, S. M. van den Heiligenberg, S. D. Bakker, A. N. M. Wymenga, I. M. Oving, R. M. Bijlsma, P. J. van Diest, J. B. Vermorken, H. van Tinteren, S. C. Linn

**Affiliations:** 1 Department of Molecular Pathology, Netherlands Cancer Institute, Plesmanlaan 121, 1066 CX Amsterdam, Netherlands; 2 Biometrics Department, Netherlands Cancer Institute, Amsterdam, Netherlands; 3 Medical Affairs, Agendia NV, Amsterdam, Netherlands; 4 Department of Medical Oncology, Amsterdam UMC, Amsterdam, Netherlands; 5 Department of Medical Oncology, Sint-Antonius Hospital, Nieuwegein, Netherlands; 6 Department of Internal Medicine, Streekziekenhuis Koningin. Beatrix, Winterswijk, Netherlands; 7 Department of Surgery, Diakonessenhuis, Utrecht, Netherlands; 8 Department of Medical Oncology, OLVG, Amsterdam, Netherlands; 9 Department of Internal Medicine, Red Cross Hospital, Beverwijk, Netherlands; 10 Department of Medical Oncology, Spaarne Gasthuis, Hoofddorp, Netherlands; 11 Department of Medical Oncology, Dijklander Ziekenhuis, Hoorn, Netherlands; 12 Department of Internal Medicine, Zaans Medisch Centrum, Zaandam, Netherlands; 13 Department of Medical Oncology, Medisch Spectrum Twente, Enschede, Netherlands; 14 Department of Medical Oncology, Ziekenhuisgroep Twente, Almelo, the Netherlands; 15 Department of Medical Oncology, UMCU, Utrecht, Netherlands; 16 Department of Pathology, UMCU, Utrecht, Netherlands; 17 Department of Medical Oncology, University Hospital Antwerp (UZA), Edegem, Belgium; 18 Faculty of Medicine and Health Sciences, University of Antwerp, Antwerp, Belgium; 19 Trial and Data Center, Princess Maxima Centre for Pediatric Oncology, Heidelberglaan 25, 3584 CS Utrecht, The Netherlands; 20 Department of Medical Oncology, Netherlands Cancer Institute, Plesmanlaan 121, 1066 CX Amsterdam, Netherlands

**Keywords:** Early breast cancer, Postmenopausal, MammaPrint 70-gene signature, Prognosis, Endocrine treatment, Overtreatment

## Abstract

**Purpose:**

Guidelines recommend endocrine treatment for estrogen receptor-positive (ER+) breast cancers for up to 10 years. Earlier data suggest that the 70-gene signature (MammaPrint) has potential to select patients that have an excellent survival without chemotherapy and limited or no tamoxifen treatment. The aim was to validate the 70-gene signature ultralow-risk classification for endocrine therapy decision making.

**Methods:**

In the IKA trial, postmenopausal patients with non-metastatic breast cancer had been randomized between no or limited adjuvant tamoxifen treatment without receiving chemotherapy. For this secondary analysis, FFPE tumor material was obtained of ER+HER2− patients with 0–3 positive lymph nodes and tested for the 70-gene signature. Distant recurrence-free interval (DRFI) long-term follow-up data were collected. Kaplan–Meier curves were used to estimate DRFI, stratified by lymph node status, for the three predefined 70-gene signature risk groups.

**Results:**

A reliable 70-gene signature could be obtained for 135 patients. Of the node-negative and node-positive patients, respectively, 20% and 13% had an ultralow-risk classification. No DRFI events were observed for node-negative patients with an ultralow-risk score in the first 10 years. The 10-year DRFI was 90% and 66% in the low-risk (but not ultralow) and high-risk classified node-negative patients, respectively.

**Conclusion:**

These survival analyses indicate that the postmenopausal node-negative ER+HER2− patients with an ultralow-risk 70-gene signature score have an excellent 10-year DRFI after surgery with a median of 1 year of endocrine treatment. This is in line with published results of the STO-3-randomized clinical trial and supports the concept that it is possible to reduce the duration of endocrine treatment in selected patients.

**Supplementary Information:**

The online version contains supplementary material available at 10.1007/s10549-022-06618-z.

## Background

Endocrine therapy is a key element of adjuvant systemic treatment for patients with estrogen receptor-positive (ER+) breast cancer, and guidelines recommending endocrine therapy for up to 10 years [1]. Five years of tamoxifen reduces 10-year breast cancer mortality rates by approximately 25% (proportionally) compared with no endocrine therapy, while this is 40% (proportionally) for five years of an aromatase inhibitor (AI) compared to no endocrine therapy [2]. The absolute 15-year breast cancer mortality reduction of 5 years of adjuvant tamoxifen versus nil is 9.2% [3]. Extension of adjuvant endocrine therapy duration for up to 10 years further reduces recurrence risk, with hazard rates of several randomized controlled trials varying between 0.57 and 1.0, depending on tamoxifen and aromatase inhibitor sequence, total endocrine therapy duration in the control arm, and case mix of the population under study [1]. Survival benefits have been observed for 10 versus 5 years of tamoxifen and for 5 years of AI therapy after 5 years of tamoxifen [1]. The absolute 15-year breast cancer mortality reduction for 10 versus 5 years of tamoxifen is 2.8% [4]. Longer follow-up of the extended-AI therapy trials will answer the question of whether longer AI therapy duration will translate into a survival benefit [1].

Although endocrine therapy toxicities are rarely life threatening, side effects are common and are leading to non-adherence which in turn can lead to a higher recurrence risk. Non-adherence is more frequent in women experiencing a moderate to high impact of the side effects on their daily lives [5, 6]. Common side effects include hot flashes, muscle and joint pain, weight gain, fatigue, mood swings, difficulty concentrating, numbness or tingling in the extremities, vaginal dryness, and hair loss. Low self-esteem and low libido are perhaps more a consequence of the above-mentioned side effects but, if present, can have a considerable impact on patients’ lives [5, 6]. Rare, life-threatening tamoxifen-related side effects include thromboembolic complications and, restricted to postmenopausal women, an increased endometrial cancer risk [7]. AI-specific side effects are osteoporosis with increased fracture risk, and increased odds of cardiovascular events [2, 8].

A test identifying patients who have an excellent prognosis with only a limited duration of endocrine therapy would allow us to reduce overtreatment and improve patients’ quality of life. On the other hand, such a test might help increase treatment adherence in patients for whom longer endocrine treatment is required to substantially increase the odds of a good outcome.

The Food & Drug Administration (FDA)-cleared 70-gene signature MammaPrint was originally developed to identify patients who have a low-risk of distant recurrence and cancer-related death and who may be candidates for (neo)adjuvant chemotherapy de-escalation [9–11].

More recently, a threshold identifying an ultralow-risk group was developed on the same 70-gene signature and validated in a post hoc analysis of patients from the STO-3 trial. The ultralow-risk group consists of patients with indolent tumors and excellent prognosis up to 20 years after diagnosis [12, 13]. The STO-3 trial included node-negative patients, with tumors up to 30 mm, who did not receive chemotherapy and only limited (2 or 5 years) or no tamoxifen treatment. The 15% of patients in the STO-3 study with an ultralow-risk classification had a 20-year breast cancer-specific survival of 97% with limited endocrine treatment and 94% without any endocrine treatment, with no statistically significant difference between the two groups.

These data can have a high impact and help de-escalation of endocrine treatment in a substantial group of breast cancer patients; therefore, we set out to validate these data in an independent dataset. The Dutch IKA trial dataset was used for the validation [14, 15], which consists of well-annotated, high-quality data and tumor tissue blocks of patients treated with no or only a short duration of adjuvant endocrine therapy and 20 years of follow-up available.

Our goal was to validate the association of the 70-gene signature ultralow-risk classification with indolent behavior and excellent prognosis in patients with ER+HER2− invasive, node-negative, and node-positive breast cancer treated with 0–3 years of tamoxifen in the IKA-randomized clinical trial.

## Methods

### Patients and material

For the current analysis, we used a subset of the Dutch multicenter IKA-randomized clinical trial that enrolled patients from 1982 till 1994. The REMARK (Reporting Recommendations for Tumor Marker Prognostic Studies) criteria were used to report this study [16]. Postmenopausal, non-metastatic, breast cancer patients (*N* = 1662) had been randomized between no adjuvant endocrine therapy (control arm), 1 or 3 years of tamoxifen treatment. The patient characteristics for the full-study population have been presented previously, and clinical outcome data were part of the Oxford meta-analysis [14, 17, 18]. Based on an interim analysis in 1989, the study protocol was amended for all node-positive patients to receive at least one year of tamoxifen. None of the patients had received (neo)adjuvant chemotherapy, as was the standard at that time for postmenopausal patients. Only local therapy was mandatory (mastectomy/breast conserving surgery ± radiotherapy, or upfront curative radiotherapy without surgery for patients with tumor-positive sub-clavicular ipsilateral lymph nodes (*N* = 55/1662 (3.3%)).

Formalin-fixed paraffin-embedded (FFPE) tumor blocks were available for 739 patients who had received upfront surgery. Estrogen receptor alpha (ER), progesterone receptor (PR), HER2, Ki67, and mitotic activity index (MAI) were centrally assessed and have been reported previously [15, 18]. For the current analysis, stage I–III patients with an ER-positive, HER2-negative breast cancer were selected (*N* = 482). For 346 patients, sufficient tumor material was available to perform the 70-gene signature MammaPrint assay.

### MammaPrint RNA analysis

FFPE tumor slides (5 × 5 µm) with a minimum of 30% tumor cells were sent to Agendia for standard RNA isolation (Qiagen RNeasy FFPE kit), and 70-gene signature MammaPrint and 80-gene BluePrint Molecular Subtype testing. This was done on a custom-designed Agilent microarray according to standard protocols and with previously described thresholds [19–21]. Agendia, blinded for clinical variables and outcomes, classified the samples for 70-gene signature into three groups: ultralow, low, or high risk, and for BluePrint into three subtypes: luminal, basal, or ERBB2/HER2-type. Agendia maintains a quality system in compliance with international regulations such as the FDA and the EU in vitro diagnostics directives.

### Statistical analysis

All analyses were performed based on three 70-gene signature MammaPrint index (MPI) thresholds: ultralow risk (MPI >  + 0.355), low risk (0 > MPI ≤  + 0.355), and high risk (MPI ≤ 0) [22]. Kaplan–Meier analyses were performed for recurrence-free interval (RFI), distant recurrence-free interval (DRFI), and breast cancer-specific survival (BCSS) according to the DATECAN definitions [23]. Survival distributions were compared using the log-rank test. In a multivariable cox proportional hazard model adjusting for age, PR, T-stage, grade, allocated treatment arm, and stratified for nodal status and study protocol version, the hazard ratios per 70-gene signature risk group are calculated with the ultralow-risk as reference. A likelihood ratio test on the multivariable model predicting breast cancer-specific survival with or without 70-gene signature risk was used to test for added value to the known prognostic variables. For risk groups that have equal RFI and DRFI events, we report survival estimates as (D)RFI for simplicity. For all endpoints, patients were censored at diagnosis of a new primary cancer. In case of an ipsilateral tumor, local assessment was followed for the classification as recurrence or new primary tumor. Deaths with unknown cause after distant breast cancer recurrence were classified as BCSS events, similar to the approach taken in the Oxford Overview [17].

The survival tree developed using recursive partitioning in the STO-3 trial was also followed for the node-negative patients in the current study, resulting in four groups [13]. For the node-positive patients, a new decision tree was created using the rpart package (version 4.1–15) in R (version 3.6.1). The following input variables were used: 70-gene signature risk, age, Ki67, MAI, T-status, and grade. The final tree was selected with a tenfold cross validation.

## Results

**Fig. 1:**
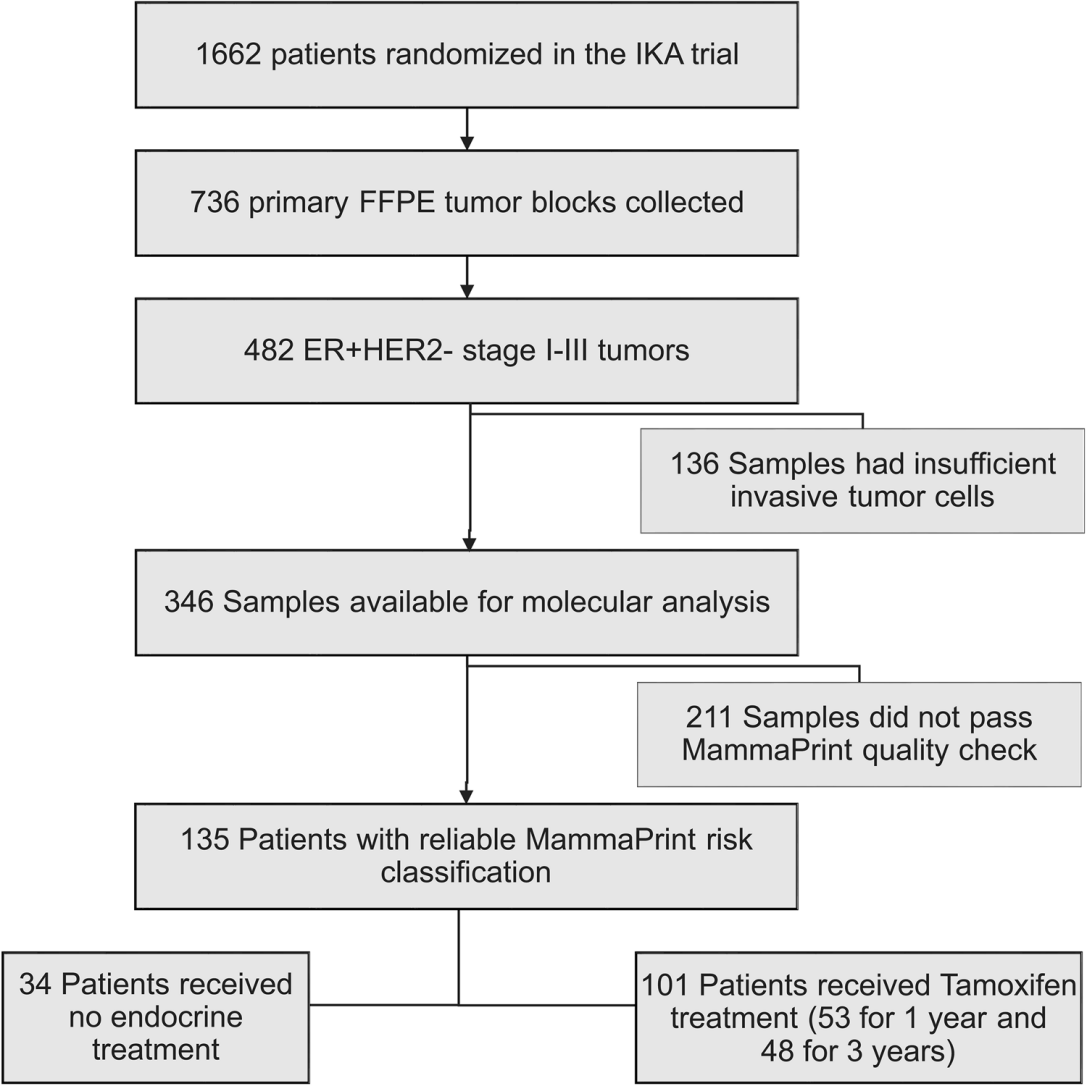
CONSORT flow diagram of the IKA-randomized clinical trial patients used for analysis. *FFPE* formalin-fixed paraffin embedded, ER+ estrogen receptor positive, *HER2−* human epidermal growth factor receptor 2 negative

A 70-gene signature score was obtained for 135 ER+, HER2− samples, of which 34 patients received no adjuvant endocrine treatment at all (Fig. 1). 101 patients were randomized to receive 1 year (*N* = 53) or 3 years (*N* = 48) of adjuvant tamoxifen and no other systemic treatment for the primary tumor. Due to a change in randomization after the interim analysis (see methods), node-positive and larger tumors are enriched in the tamoxifen-treated arm (Table 1). No significant selection differences were detected between 135 patients with ER+HER2− tumors with a 70-gene signature result and the 347 patients with ER+HER2− tumors without a 70-gene signature result for the most important tumor and patient characteristics (Table 1).

**Table 1:** Patient characteristics of the 135 patients with 70-gene MammaPrint results and assigned randomization group compared to the ER+HER2− stage I–III patients of the IKA trial that were not analyzed. *P* values calculated between no tamoxifen and 1-3 year tamoxifen. Missing cases are not included in the calculation of the *p* values or percentages. No differences were statistically significant in the comparison between 70-gene signature tested and not tested ER+HER2- patients (*p *values not shown). Bold *p* values are below 0.05

	No tamoxifen *N* = 34	1–3 year tamoxifen *N* = 101	*p* value	Not tested ER+HER2− *N* = 347
Year of diagnosis				
1982–1989	11 (32%)	37 (37%)		114 (33%)
1989–1994	23 (68%)	64 (63%)		233 (67%)
Age (years)				
< 55	0	9 (9%)	0.12	23 (7%)
55–64	19 (56%)	42 (41%)		141 (41%)
Surgery				
Breast conserving	15 (44%)	32 (32%)	0.27	104 (30%)
Mastectomy	19 (56%)	69 (68%)		238 (70%)
Nodal status				
Negative	29 (85%)	51 (50%)	**0.001**	194 (56%)
Positive	5 (15%)	50 (50%)		153 (44%)
PR*				
< 10%	14 (47%)	42 (45%)	0.46	161 (50%)
≥ 10–50%	5 (17%)	25 (27%)		50 (16%)
> 50%	11 (37%)	26 (28%)		110 (34%)
Ki67*				
< 5%	18 (67%)	64 (74%)	0.50	183 (69%)
≥ 5–10%	7 (26%)	14 (16%)		51 (19%)
> 10%	2 (7%)	9 (10%)		32 (12%)
MAI*				
< 8/2 mm^2^	18 (55%)	55 (55%)	1.00	186 (54%)
≥ 8/2 mm^2^	15 (45%)	45 (45%)		157 (46%)
Grade				
1	13 (38%)	32 (32%)	0.80	103 (30%)
2	11 (32%)	38 (38%)		139 (40%)
3	10 (29%)	31 (31%)		103 (30%)
T-size*				
T1	14 (42%)	21 (21%)	**0.02**	114 (33%)
T2	19 (58%)	70 (70%)		204 (59%)
T3	0	9 (9%)		29 (8%)
Randomization				
No endocrine treatment	34 (100%)	0	**< 0.001**	82 (24%)
1 year of tamoxifen	0	53 (53%)		174 (50%)
3 years of tamoxifen	0	48 (48%)		91 (26%)
Tamoxifen received^#^				
Median duration [Q1–Q3]	0 [0–0]	1.3 [1.0–3.0]	**< 0.001**	1.1 [1.0–3.0]

The enrichment for node-positive patients in the 1–3 year tamoxifen-treated arm is due to the amendment in 1989

*For PR, Ki67, Mitotic Activity Index (MAI), and T-status some cases are missing (respectively, 12, 21, 2, and 2) of the tested patients

^#^*p* value for tamoxifen duration difference was calculated with Kruskal–Wallis test

Using the 70-gene signature, we classified 53 (40%), 59 (43%), and 23 (17%) of the patients as respectively high, low, and ultralow risk. In Table 2, the distributions of clinicopathological characteristics within the three 70-gene signature scores are shown. MAI, PR status, and grade were significantly different between the groups: In the ultralow-risk group, 13% (3/23) of the patients had a tumor with MAI ≥ 8/2 mm2, while this was 35% (20/57) and 70% (37/53) for the genomic low and high-risk group, respectively. The PR status was negative (< 10% positive staining) in 29% (6/21) of ultralow-risk tumors, while this was 44% (23/52) and 54% (27/50) for the genomic low-risk and high-risk tumors, respectively. Similarly, in the ultralow-risk group, 65% (15/23) of the tumors were grade I, while only 39% (23/59) and 13% (7/53) were grade I for the genomic low and high-risk tumors, respectively. Within the node-negative subset, 20% (16/80) patients had an ultralow-risk 70-gene signature result. All ultralow and low-risk patients had a Luminal classification by BluePrint, whereas 94% (50/53) of the high-risk tumors were classified as Luminal.

**Table 2:** 70-gene signature classification and patient characteristics divided by the MammaPrint risk score. Bold *p* values are below 0.05

	Ultralow-risk *N* = 23	Low-risk *N* = 59	High-risk *N* = 53	*p* value
Year of diagnosis				
1982–1989	8 (35%)	20 (34%)	20 (38%)	0.91
1989–1994	15 (65%)	39 (66%)	33 (62%)	
Age (years)				
< 54	2 (9%)	4 (7%)	3 (6%)	0.85
55–64	10 (44%)	24 (41%)	27 (51%)	
> 65	11 (48%)	31 (53%)	23 (43%)	
Surgery				
Breast conserving	12 (52%)	17 (29%)	18 (34%)	0.14
Mastectomy	11 (48%)	42 (71%)	35 (66%)	
Nodal status				
Negative	16 (70%)	33 (56%)	31 (58%)	0.52
Positive	7 (30%)	26 (44%)	22 (42%)	
PR*				
< 10%	6 (29%)	23 (44%)	27 (54%)	**0.04**
≥ 10–50%	3 (14%)	14 (27%)	13 (26%)	
> 50%	12 (57%)	15(29%)	10 (20%)	
Missing	2	7	3	
Ki67*				
< 5%	14 (64%)	32 (68%)	36 (80%)	0.05
≥ 5–10%	8 (36%)	8 (17%)	5 (11%)	
> 10%	0	7 (15%)	4 (9%)	
Missing	1	12	8	
MAI*				
< 8/2 mm^2^	20 (87%)	37 (65%)	16 (30%)	**< 0.001**
≥ 8/2 mm^2^	3 (13%)	20 (35%)	37 (70%)	
Missing	0	2	0	
Grade*				
1	15 (65%)	23 (39%)	7 (13%)	**< 0.001**
2	8 (35%)	22 (37%)	19 (36%)	
3	0	14 (24%)	27 (51%)	
T-size*				
T1	4 (17%)	19 (33%)	12 (23%)	0.41
T2	17 (74%)	34 (59%)	38 (73%)	
T3	2 (9%)	5 (9%)	2 (4%)	
Randomization				
No treatment	5 (22%)	13 (22%)	16 (30%)	0.59
1 year tamoxifen	10 (43%)	21 (36%)	22 (42%)	
3 years tamoxifen	8 (35%)	25 (42%)	15 (28%)	
Tamoxifen received^a^				
Median duration [Q1–Q3]	1.1 [0.9–3.1]	1.1 [0.5–3.0]	1.0 [0–2.0]	0.12
Second primary tumor				
Number of events	2 (9%)	6 (10%)	6 (11%)	1.00

^a^For tamoxifen duration Kruskal–Wallis test was used

^*^For PR, Ki67, Mitotic Activity Index (MAI) and T-size the missing cases are not included in the calculation of *p* values or percentages

### Survival analysis

**Fig. 2:**
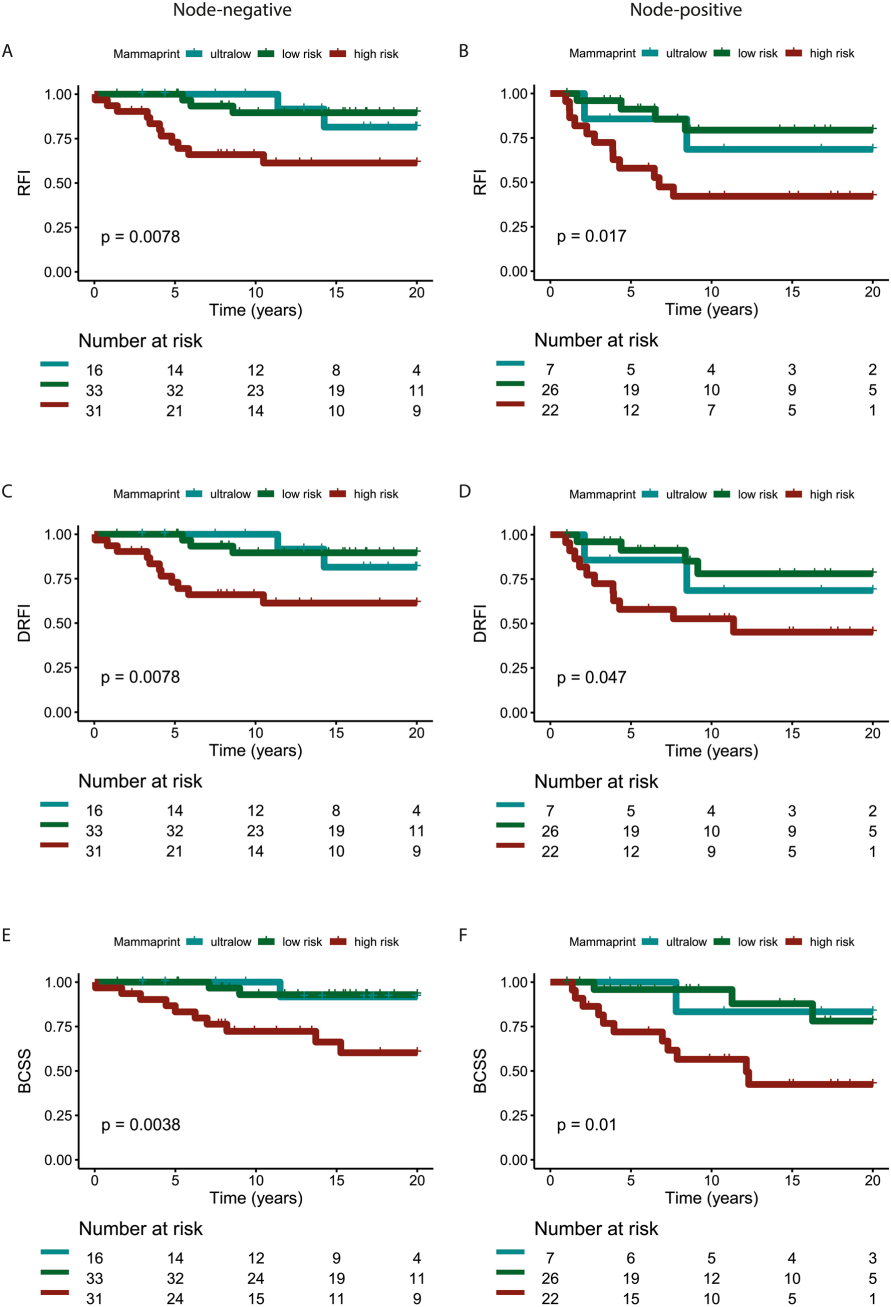
Kaplan–Meier plots of survival in node-negative and node-positive patients. Recurrence-Free Interval of the patients stratified based on 70-gene MammaPrint risk score for **A** node-negative patients and **B** node-positive patients. Distant Recurrence-Free Interval of the patients stratified based on 70-gene MammaPrint risk score for **C** node-negative patients and **D** node-positive patients. Breast cancer-specific survival of the patients stratified based on 70-gene MammaPrint risk score for **E** node-negative patients and **F** node-positive patients

Median follow-up was 27 years using the reverse Kaplan–Meier method [24]. In the node-negative patients with an ultralow-risk 70-gene signature score (*N* = 16), no (D)RFI events were observed in the first 10 years after diagnosis, while the 20-year (D)RFI was 82% [95% CI 61–100] (Table 3). For the 70-gene signature low-risk (but not ultralow) node-negative patients, the 10-year and 20-year (D)RFI were 90% [95% CI 79–100] for both time points. In the genomic high-risk node-negative patients, the 10-year (D)RFI was 66% [95% CI 51–86], and the 20-year (D)RFI was 61% [95% CI 45–83] (Table 3). The corresponding Kaplan–Meier plots for node-negative patients are shown in Fig. 2A (RFI) and Fig. 2C (DRFI). Breast cancer-specific survival is shown in Fig. 2E for the node-negative patients. The 10-year BCSS in node-negative patients is 100% for the ultralow-risk group, 93% [95% CI 84–100] for the low-risk group, and 72% [95% CI 58–91] for the 70-gene signature high-risk group. The 20-year BCSS in node-negative patients is respectively for ultralow, low, and high risk: 92% [95% CI 77–100], 93% [95% CI 84–100], and 60% [95% CI 43–85].

**Table 3:** Survival rates by 70-gene signature risk classification with 95% confidence intervals for recurrence-free interval, distant recurrence-free interval, and breast cancer-specific survival at 10, 15 and 20 years split by nodal status

MammaPrint	*N*	Survival	10 years	15 years	20 years
Node-negative	16				
Ultralow-risk		RFI	100%	82% [61–100]	82% [61–100]
		DRFI	100%	82% [61–100]	82% [61–100]
		BCSS	100%	92% [77–100]	92% [77–100]
Low-risk	33	RFI	90% [79–100]	90% [79–100]	90% [79–100]
		DRFI	90% [79–100]	90% [79–100]	90% [79–100]
		BCSS	93% [84–100]	93% [84–100]	93% [84–100]
High-risk	31	RFI	66% [51–86]	61% [45–83]	61% [45–83]
		DRFI	66% [51–86]	61% [45–83]	61% [45–83]
		BCSS	72% [58–91]	66% [50–88]	60% [43–85]
Node-positive					
Ultralow-risk	7	RFI	69% [40–100]	69% [40–100]	69% [40–100]
		DRFI	69% [40–100]	69% [40–100]	69% [40–100]
		BCSS	83% [58–100]	83% [58–100]	83% [58–100]
Low-risk	26	RFI	79% [63–100]	79% [63–100]	79% [63–100]
		DRFI	78% [61–100]	78% [61–100]	78% [61–100]
		BCSS	96% [88–100]	88% [73–100]	78% [60–100]
High-risk	22	RFI	42% [25–70]	42% [25–70]	42% [25–70]
		DRFI	53% [35–79]	45% [27–75]	45% [27–75]
		BCSS	57% [39–83]	42% [24–74]	42% [24–74]

In the node-positive patients with an ultralow-risk 70-gene signature score (*N* = 7), the (D)RFI was 69% [95% CI 40–100] at 10 and 20 years. The 70-gene signature low (but not ultralow) risk node-positive patients had a RFI of 79% [95% CI 63–100] for all time points with an almost similar DRFI of 78% [95% CI 61–100]. For the high-risk node-positive patients, the 10-year and 20-year RFI was 42% [95% CI 25–70] for both time points (Table 3; Fig. 2B) and the DRFI was 53% [95% CI 35–79] at 10 years and 45% [95% CI 27–75] at 20 years (Table 3; Fig. 2D). Breast cancer-specific survival is shown in in Fig. 2F for node-positive patients. The 20-year BCSS in the node-positive patients is 83% [95% CI 58–100] for the ultralow-risk group, 78% [95% CI 60–100] for the low-risk group, and 42% [95% CI 24–74] for the 70-gene signature high-risk group (Table 3). In Online Resource 1 and 2, the BCSS without censoring for the second primary tumor are shown. The results were similar to the results with censoring for second primary tumor (Fig. 2 and Table 3).

In the first 20 years, breast cancer patients with a 70-gene high-risk tumor had a significant higher risk of disease-specific death compared with ultralow-risk patients in a multivariable Cox analysis (hazard ratio 6.35 [95% CI 1.35–29.96], while for patient with a low-, but not ultralow-risk tumor, the hazard ratio was not significant different (hazard ratio 1.27 [95% CI 0.23–6.93]. A likelihood ratio test on a multivariable model predicting breast cancer-specific survival with or without 70-gene signature risk scores indicates that the 70-gene signature risk score has added value (∆LR-*χ*^2^ = 14.6, *p* < 0.001) to the know prognostic variables.

### Survival decision tree

**Fig. 3:**
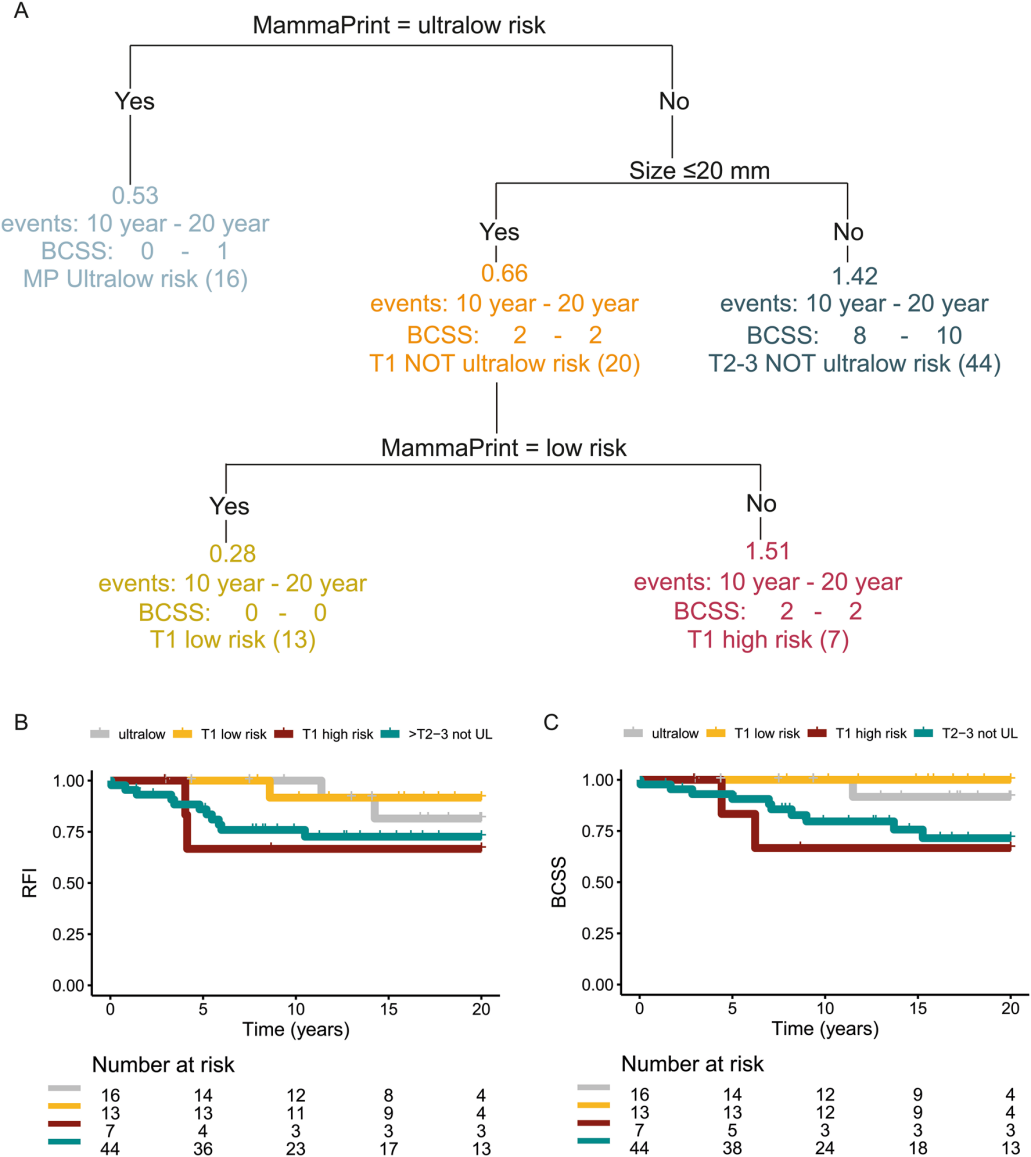
Predefined decision tree for the node-negative patients and Kaplan–Meier Plots based on the risk prediction. **A** The decision tool was proposed by Esserman and filled in with the node-negative patients from the IKA tamoxifen trial resulting in four groups. Risk prediction, number of Breast Cancer-Specific Deaths at 10 and 20 years, risk group name and number of patients are shown for each group. **B** Kaplan–Meier plots of recurrence-free interval and **C** breast cancer-specific survival are shown for the four end groups of the decision tree: MammaPrint ultralow, T1 MammaPrint low risk, T1 MammaPrint high risk, T2-3 MammaPrint not ultralow

As suggested by the recursive partitioning survival tree by Esserman et al. [13], we divided the node-negative patients first on genomic ultralow-risk and second, for the genomic non-ultralow-risk patients, on size (≤ or > 20 mm, respectively, T1 or T2–3). The T1 not ultralow-risk patients are subdivided into 70-gene signature low and high risk. The sixteen ultralow-risk patients are discussed before, and in this group, one BCSS event (6%) occurred in the 20-year follow-up. In the thirteen T1 low-risk patients, no BCSS events occurred. In the seven T1 high-risk patients, two BCSS events (29%) occurred. In the 44 T2–3 not ultralow-risk patients, ten BCSS events (23%) occurred (Fig. 3A). The Kaplan–Meier survival plots for the different groups are shown in Fig. 3B (RFI) and 3C (BCSS).

**Fig. 4:**
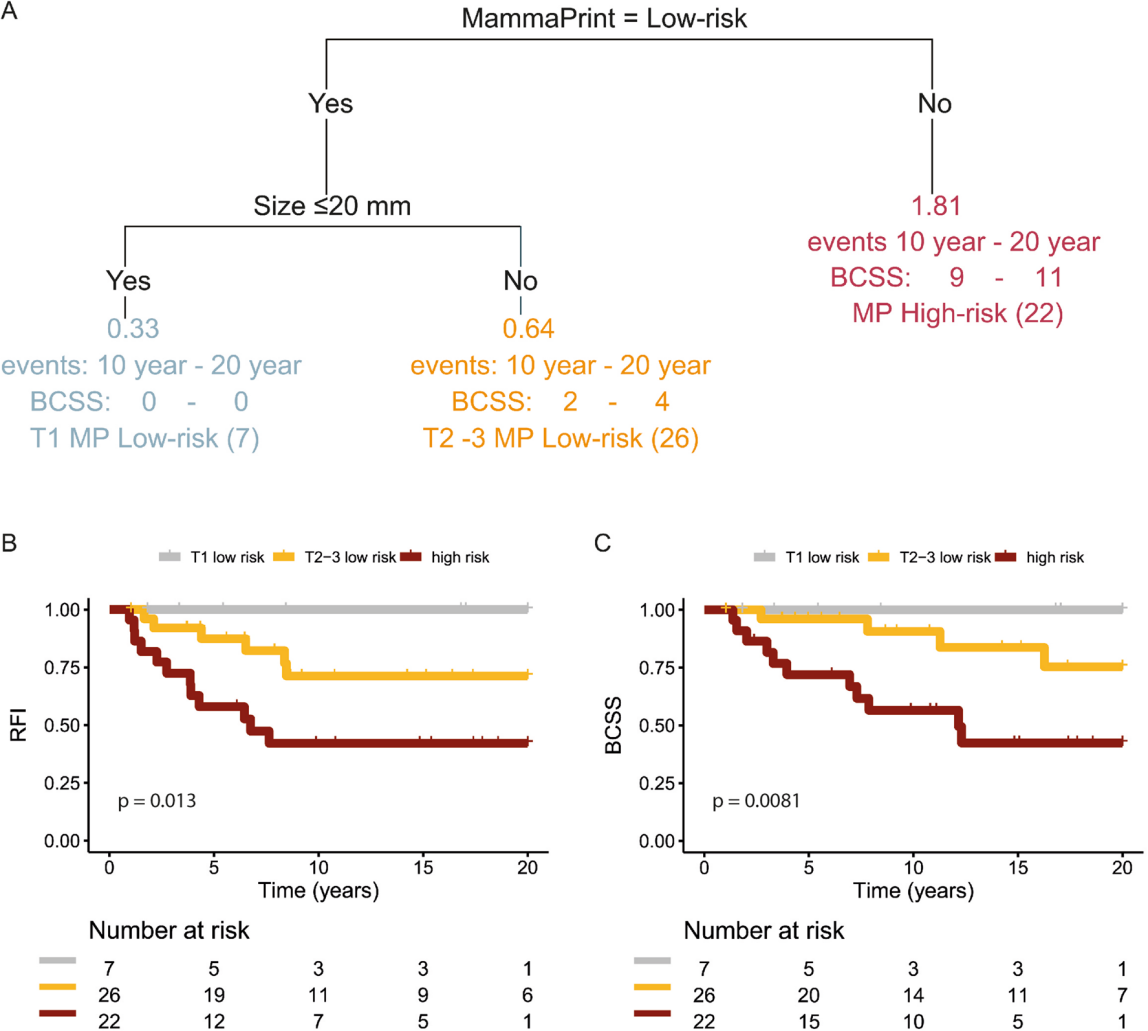
Explorative decision tree for the node-positive patients and Kaplan–Meier Plots based on the risk prediction. The decision tool was developed using rpart with input variables: 70-gene MammaPrint classification (ultralow, low risk, or high risk), age, Ki67, MAI, tumor size, and grade. **A** The node-positive patients from the IKA tamoxifen trial were classified in three groups. Risk prediction, number of Breast Cancer-Specific Deaths at 10 and 20 years, risk group name, and number of patients are shown for each group. Note that the low-risk groups include ultralow-risk patients. **B** Kaplan–Meier plots recurrence-free interval and **C** breast cancer-specific survival shown for the three final groups of the decision tree: MammaPrint low risk with size ≤ 20 mm, MammaPrint low risk with size > 20 mm, and MammaPrint high risk

For the node-positive patients, a new survival tree was made (Fig. 4) with 70-gene signature as the most informative variable to predict BCSS compared to the other input variables: age, Ki67, MAI, T-status, and grade. The 70-gene signature low-risk (including ultralow) groups are further split by size. This resulted in no BCSS event for the seven patients with 70-gene signature low risk and tumor size ≤ 20 mm. In the 70-gene signature low risk and tumor size > 20 mm, 26 patients are grouped and in this group, five BCCS events occurred (19%). In the 70-gene signature high-risk patients, nine events are observed in 22 patients (41%).

## Discussion

In this post hoc analysis of a representative subset of postmenopausal, stage I-III patients with ER+HER2− tumor that participated in the randomized IKA trial of adjuvant tamoxifen versus nil, we observed an excellent outcome with no BCSS events during the first 10 years for node-negative patients with a ER+HER2− and 70-gene signature ultralow-risk tumor. These data confirm earlier findings for the ultralow-risk 70-gene signature result in the STO-3 trial [13] and are in line with the recent findings in the MINDACT series [25]. Importantly, in our studies, we did not only report on BCSS events but also on any type of breast cancer recurrence (RFI). Remarkably, node-negative, postmenopausal patients with ER+HER2− ultralow-risk tumors did not recur at all in the first 10 years of follow-up. On the other hand, our data also show that postmenopausal patients with ultralow-risk tumors still have a risk of recurrence after 10 years if treated with only 0–3 years of adjuvant tamoxifen. The mild discrepancy in the prognosis of the ultralow-risk patients between the IKA trial and the STO-3 trial after 10 years might be caused by noteworthy differences in the patient case mixes. Most importantly, the IKA trial was not restricted to patients with a tumor size < 30 mm as in the STO-3 trial [13]. The IKA trial included both lymph node-negative and lymph-positive patients, and in the IKA post hoc analysis, we selected only ER+HER2− patients, while in the STO-3 trial also, ER+ HER2 + and ER-HER2 − patients were included in the secondary analysis. In addition, our post hoc study is five times smaller than the STO-3 secondary study, resulting in less than ten ultralow-risk patients at risk after 15 years, which makes long-term estimates imprecise. Of note, when focusing on the 10-year and 15-year BCSS data, our data are very similar to the STO-3 data [13].

In the STO-3 trial, 15% of node-negative ER+ HER2 any patients and 19% of the ER+HER2− patients had an ultralow-risk tumor [13], which is very close to the 20% found in our study. Importantly, both trials mainly ran in an era before the introduction of mammography screening, and hence, current incidence of node-negative, ER+HER2− ultralow-risk tumors among postmenopausal women may even be higher, as earlier observed for 70-gene signature low-risk tumors [26].

The recursive partitioning survival tree [13] analyses of our study and the STO-3 study may help guide postmenopausal, node-negative, HER2-negative patients who experience a substantial deterioration in the quality of life while taking adjuvant endocrine therapy. In the case of an ultralow-risk tumor smaller than or equal to 30 mm in size or a low-risk tumor less than or equal to 20 mm in size, abandoning adjuvant endocrine therapy early will only have a marginal impact on long-term breast cancer-specific survival. On the other hand, for patients with a low-risk but no ultralow-risk tumor over 20 mm in size, or for patients with a high-risk tumor, the substantial long-term recurrence risk may motivate these patients to adhere to their endocrine therapy. Repeatedly, studies have shown that around 10–30% of patients stop adjuvant endocrine therapy early or do not start at all [7]. This also implicates that the true benefit of adjuvant endocrine therapy is larger than observed in meta-analyses of randomized trials that generally use the intention-to-treat principle [7, 27].

In addition, currently, aromatase inhibitors have predominantly replaced tamoxifen as adjuvant endocrine therapy in the postmenopausal setting, leading to a further reduction in recurrence risk [1]. Since low MAI (< 8/2 mm^2^) has recently been reported to be a useful biomarker for endocrine therapy sensitivity [15], and in our study, 87% of ultralow-risk tumors had a low MAI, while only 30% of high-risk tumors had a low MAI, it is highly likely that already 1–3 years of adjuvant endocrine therapy will substantially reduce the already limited risk of breast cancer recurrence in ultralow-risk, node-negative, postmenopausal patients. In the same vein, patients with ER+ HER− ultralow-risk tumors are enriched for PR positivity. While PR positivity has been associated with tamoxifen sensitivity, the absolute breast cancer recurrence risk for ultralow-risk patients is already very low, resulting in a very small absolute benefit from tamoxifen treatment.

Besides 70-gene signature, several other multigene prognostic tests have been developed, and some of these have entered daily clinical practice [28]. Most evidence has been compiled for these tests to be used for the decision to forego adjuvant chemotherapy in node-negative, ER+HER2− postmenopausal patients (e.g., Oncotype DX, MammaPrint, Endopredict, Prosigna, and Breast Cancer Index). Most secondary analyses of large randomized clinical trials, such as ATAC, ABCSG6, ABCSG8, and STO-3 have been instrumental in demonstrating clinical utility [29–33]. Only for MammaPrint, 70-gene signature and Oncotype DX have level I evidence been generated with prospective, randomized clinical trials that also included premenopausal women [11, 34]. Secondary analyses of the STO-3 trial have indicated that the70-gene signature and Breast Cancer Index multigene tests may help in selecting node-negative, postmenopausal ER+ HER- breast cancer patients for whom limited or no adjuvant tamoxifen would suffice [13, 33]. Data presented here further support the MammaPrint 70-gene signature for this indication. Moreover, a prospective trial to further validate this finding seems unrealistic in this group of patients, due to the low event rate and the long follow-up that will be needed.

In patients with ER+HER2− node-positive breast cancer, the 70-gene signature predicts BCSS difference between genomic high risk and low risk (including the ultralow-risk), but not between ultralow-risk and low-risk patients. In an exploratory analysis, the second variable predicting BCSS in this group of patients is tumor size (below or above 20 mm), resulting in no BCSS event out of seven patients in the 70-gene low risk (including ultralow) and tumor size < 20 mm group. Although the median follow-up in this group is short (5 years) and the numbers are small, there is a significant survival difference between the groups. It would be very interesting to see if this result can be confirmed in other series with node-positive patients treated without chemotherapy and only limited endocrine therapy.

Limitations of this study include the small sample size. Only 736 of the 1662 (44%) patients were subtyped and for 72% (346/482) of the ER+HER2− patients sufficient left-over material was available. Furthermore, due to the use of old FFPE material, 61% (211/346) of the samples failed the quality criteria of the 70-gene signature. There were no differences between patients with tumors that resulted in a 70-gene signature score and the remaining ER+HER2− patients, reassuring that there was no obvious selection bias. Furthermore, at the time of the study, tamoxifen was used as standard of care, while nowadays, treatment with an aromatase inhibitor (AI) is more common in postmenopausal patients. However, it is expected that ultralow-risk patients with no or only limited tamoxifen treatment would also have an excellent prognosis with no or limited AI treatment. Finally, we lacked information on tamoxifen treatment non-compliance that generally ranges between 10 and 30% [7]. However, non-compliance would result in underestimating outcome, meaning that with 100% compliance, tamoxifen-treated patients would even have a better outcome than reported here.

Strengths of this study include the uniqueness of well-annotated data of patients participating in a trial randomized between no treatment or only a short duration of adjuvant endocrine therapy and with 20 years of follow-up data available.

In conclusion, an ultralow-risk MammaPrint 70-gene signature test result can help avoid systemic overtreatment in postmenopausal patients with node-negative, estrogen receptor-positive, HER2-negative breast cancer and has the potential to improve shared decision making by clinicians and patients regarding limited or no adjuvant endocrine therapy.

## Supplementary Information

Below is the link to the electronic supplementary material.

Supplementary file1 Online Resource 1. Kaplan-Meier Plots of Breast Cancer-Specific Survival in node-negative and node-positive patients without censoring second tumor. Breast cancer-specific survival of the patients stratified based on 70-gene MammaPrint risk score for (A) node-negative patients and (B) node-positive patients. (PDF 148 kb)

Supplementary file2 Online Resource 2. Table with survival rates by 70-gene signature risk classification with 95% confidence intervals for Breast Cancer-Specific Survival at 10, 15 and 20 years split by nodal status without censoring for second tumor. (DOCX 12 kb)

Supplementary file3 Online Resource 3. Data file of all 135 patients with 70-gene signature, clinicopathological characteristics and survival data. (CSV 22 kb)

## Data Availability

The data that support the findings of this study can be found in supplement file S3. Other data are available from the authors upon reasonable request and with permission of The Netherlands Institute.
