# Supplementary material for: Limiting systemic endocrine overtreatment in postmenopausal breast cancer patients with an ultralow classification of the 70-gene signature
Source: Breast Cancer Res Treat. 2022 May 19;194(2):265–78. doi: 10.1007/s10549-022-06618-z (PMC9239940; doi:10.1007/s10549-022-06618-z)
Supplement: Supplementary file 1 — Supplementary file1 Online Resource 1. Kaplan-Meier Plots of Breast Cancer-Specific Survival in node-negative and node-positive patients without censoring second tumor. Breast cancer-specific survival of the patients stratified based on 70-gene MammaPrint risk score for (A) node-negative patients and (B) node-positive patients. (PDF 148 kb) [file 10549_2022_6618_MOESM1_ESM.pdf]

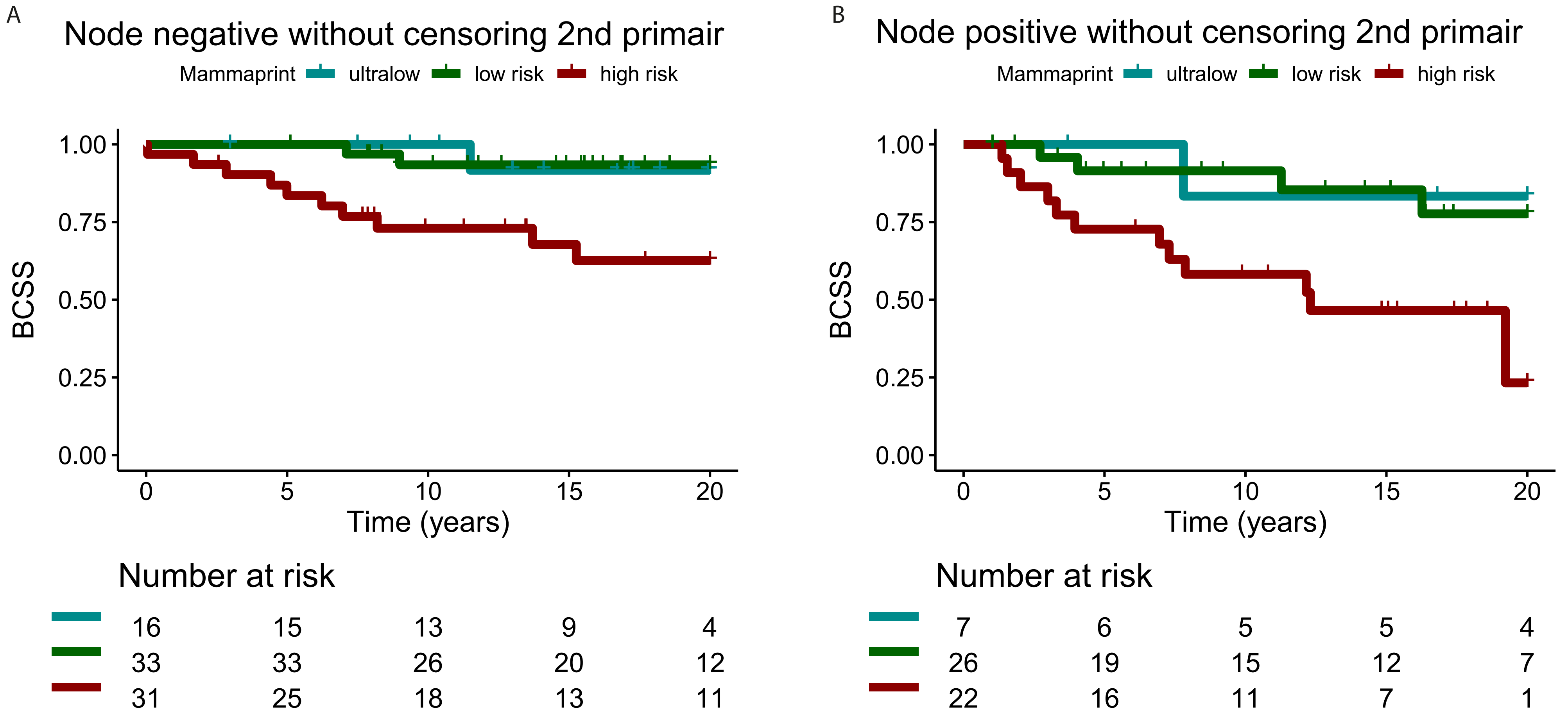
A
Node negative without censoring 2nd primair
Mammaprint ultralow low risk high risk
BCSS
1.00
0.75
0.50
0.25
0.00
0 5 10 15 20
Time (years)
Number at risk
16 15 13 9 4
33 33 26 20 12
31 25 18 13 11
B
Node positive without censoring 2nd primair
Mammaprint ultralow low risk high risk
BCSS
1.00
0.75
0.50
0.25
0.00
0 5 10 15 20
Time (years)
Number at risk
7 6 5 5 4
26 19 15 12 7
22 16 11 7 1
