# Supplementary material for: Limiting systemic endocrine overtreatment in postmenopausal breast cancer patients with an ultralow classification of the 70-gene signature
Source: Breast Cancer Res Treat. 2022 May 19;194(2):265–78. doi: 10.1007/s10549-022-06618-z (PMC9239940; doi:10.1007/s10549-022-06618-z)
Supplement: Supplementary file 2 — Supplementary file2 Online Resource 2. Table with survival rates by 70-gene signature risk classification with 95% confidence intervals for Breast Cancer-Specific Survival at 10, 15 and 20 years split by nodal status without censoring for second tumor. (DOCX 12 kb) [file 10549_2022_6618_MOESM2_ESM.docx]

**Supplementary Table S2** Survival rates by 70-gene signature risk classification with 95% confidence intervals for Breast Cancer Specific Survival at 10, 15 and 20 years split by nodal status without censoring for second tumor.

|  | **MammaPrint** | ***N*** | **Survival** | **10 years** | **15 years** | **20 years** |
| --- | --- | --- | --- | --- | --- | --- |
| **Node-negative** | Ultralow | 16 | BCSS | 100% | 92% [77 – 100] | 92% [77 – 100] |
|  | Low-risk | 33 | BCSS | 93% [85 -100] | 93% [85 – 100] | 93% [85 – 100] |
|  | High-risk | 31 | BCSS | 73% [59 – 91] | 68% [52 – 88] | 63% [46 – 85] |
|  |  |  |  |  |  |  |
|  | **MammaPrint** | ***N*** | **Survival** | **10 years** | **15 years** | **20 years** |
| **Node-positive** | Ultralow | 7 | BCSS | 83% [58 -100] | 83% [58 – 100] | 83% [58 – 100] |
|  | Low-risk | 26 | BCSS | 91% [81 -100] | 85% [71 – 100] | 78% [60 – 100] |
|  | High-risk | 22 | BCSS | 58% [41 – 83] | 47% [29 – 75] | 23% [5 – 100] |
